# Investigation of naturally-occurring radioactive materials (NORM) in building materials in Ireland

**DOI:** 10.1140/epjs/s11734-026-02353-4

**Published:** 2026-05-13

**Authors:** R. E. Djabou, M. Dodek, C. Sainz, I. Fuente, L. L. Vintró, O. Hanley, J. Goggins, M. Foley

**Affiliations:** 1 Physics, School of Natural Sciences, University of Galway, Galway, Ireland; 2 Radon Group, University of Cantabria, C/Cardenal Herrera Oria S/N, 39011 Santander, Spain; 3 School of Physics, University College Dublin, Dublin, Ireland; 4 Environmental Protection Agency, Dublin, Ireland; 5 School of Engineering, College of Science and Engineering, University of Galway, Galway, Ireland

## Abstract

Naturally-occurring radioactive materials (NORM), primarily from the decay chains of uranium (U-238) and thorium (Th-232), as well 1as potassium (K-40), are commonly found in raw materials used in the construction industry. These radionuclides contribute to indoor radiation exposure, particularly in enclosed environments. As part of the NORM-BMI project, this study evaluated the activity concentrations of Ra-226, Th-232, and K-40 in commonly used building materials in Ireland, specifically tiles, cement, and concrete. The assessment focused on calculating the Gamma Index (Iγ) to determine radiation safety, using high-resolution gamma spectrometry in three independent laboratories (University of Cantabria, University College Dublin, and the Environmental Protection Agency Laboratory). The results indicated that tile samples exhibited the highest concentrations of radionuclides, particularly Ra-226 and K-40, with Gamma Index values approaching the recommended threshold (Iγ ≈ 1). In contrast, cement and demolition concrete samples showed low activity concentrations and Gamma Index values well below 1, indicating minimal radiological risk. Comparison with European datasets confirmed the relative safety of these building materials, particularly concrete and cement. These findings support the development of national screening protocols and contribute to safer, more sustainable construction practices, while also informing EU initiatives focused on the recycling and reuse of building materials.

## Introduction

Naturally-occurring radioactive materials (NORM) are widespread in the Earth’s crust and are commonly found in raw materials used to produce building and construction products. These materials often contain radionuclides from the uranium-238 (U-238) and thorium-232 (Th-232) decay series, as well as potassium-40 (K-40). These radionuclides contribute to indoor radiation exposure primarily through gamma radiation and through the inhalation of radon gas.

The radiological impact of NORM becomes particularly significant in enclosed environments, where long-term exposure may pose health risks. According to the United Nations Scientific Committee on the Effects of Atomic Radiation (UNSCEAR), these radionuclides are a major contributor to the natural background radiation to which humans are continuously exposed [1]. The European Commission has also emphasized the importance of assessing the radiological implications of building materials, especially with the increasing use of industrial by-products and imported materials in construction [2].

Given that individuals spend approximately 80–90% of their time indoors, evaluating the contribution of building materials to indoor radiation exposure is critical for public health. Regulatory frameworks such as the EURATOM Basic Safety Standards Directive (2013/59/Euratom) [3], highlight the necessity of controlling exposure to NORM in construction contexts.

While several international studies have assessed the radiological characteristics of building materials, Ireland currently lacks a national database or standardized screening procedures [4]. The NORM-BMI project, a collaborative initiative involving the University of Galway (Ireland), the University of Cantabria (Spain), University College Dublin (Ireland), and the Environmental Protection Agency (EPA) Laboratory (Ireland), seeks to address this gap. The project aims to benchmark measurement techniques, examine variability in NORM concentrations in both newly manufactured and recycled/demolition construction materials, and explore strategies to mitigate radiological exposure.

As part of the NORM-BMI project, this study assesses NORM concentrations and calculates the Gamma Index (Iγ) for a selection of building materials commonly used in Ireland. In this phase-one study, one representative sample per material type (tiles, cement, and concrete) was selected. Each sample was independently analysed in three laboratories to assess inter-laboratory consistency, while also providing an initial indication of material variability. Accordingly, the results should be interpreted as a preliminary baseline rather than a comprehensive assessment of all building materials used in Ireland. The aim is to establish a methodological baseline, provide data to inform national policy, and contribute to broader European efforts supporting the safe and sustainable reuse of construction materials.

## Materials and methods

### Material selection

Materials were selected based on a multi-criteria framework designed to ensure scientific relevance, regulatory applicability, and logistical feasibility. First, international datasets from 29 countries were reviewed to identify building materials with documented concentrations of naturally occurring radionuclides (Ra-226, Th-232, K-40). Second, emphasis was placed on materials prevalent in the Irish construction sector, drawing on national surveys and building stock analyses [4].

Third, practical considerations such as sample availability, representativeness, and compatibility with analytical protocols were factored in. As a result, three primary material types were selected for phase one of this study: concrete, cement, and tiles. These represent both traditional and industrially processed construction materials and offer a range of mineralogical compositions suitable for assessing NORM-related radiological risks [5].

Specifically, the concrete sample was collected from demolition of a residential building constructed in 1964 in Galway, Ireland. The cement sample was a commercial CEM II type product manufactured by Irish Cement [6], representing modern industrial materials in current use. The tile sample consisted of unused ceramic tiles sourced from recent construction stock, selected to reflect newer, commercially available building materials.

### Sample preparation

A standardized protocol was adopted for all sample preparations to ensure consistency. Initial manual cleaning was performed to remove surface contaminants, including dust, coatings, and organic residues.

**Fig. 1:**
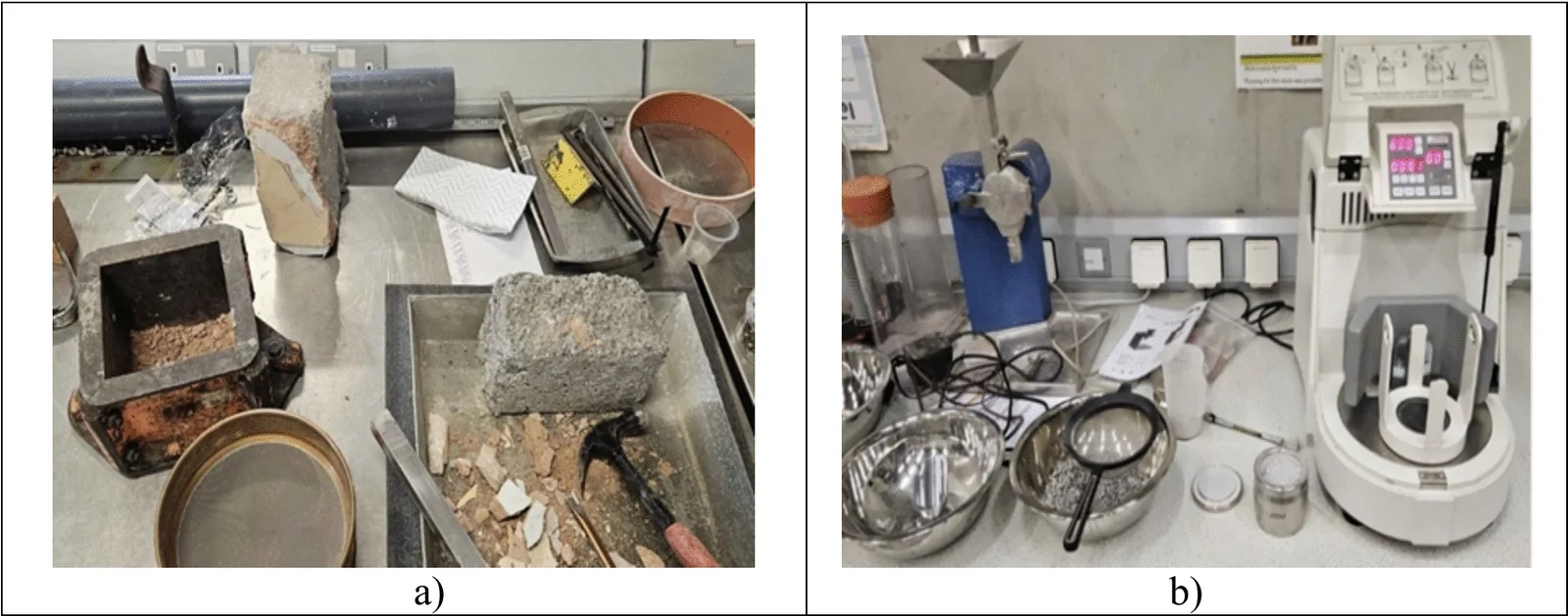
Sample preparation steps for building materials: **a** coarse crushing using a hammer, chisel and concrete compression apparatus; **b** fine pulverization using a planetary ball mill

Subsequently, the samples were mechanically crushed using hammer, chisel, and concrete compression machine to reduce them to coarse fragments approximately 3 cm in diameter, as illustrated in Fig. 1. Pulverization was then carried out using planetary ball mills to achieve a particle size of less than 1 mm, in accordance with the IAEA Technical Reports Series No. 295 [7], which recommends fine homogenization for gamma spectrometric analysis, as shown in Fig. 1.

The pulverized material was sieved to ensure uniform particle size and then transferred to sealed containers. Samples were shipped to the three participating laboratories. Upon arrival, the materials were transferred into specialized containers tailored to each laboratory’s detector geometry and sealed.

Once prepared all samples were stored for a minimum of 28 days to allow secular equilibrium to be established between Ra‑226 and its short-lived progeny Pb‑214 and Bi‑214, via Rn‑222 (half-life 3.82 days), assuming no loss of radon gas. This period is widely accepted in gamma spectrometry protocols as sufficient for using Pb‑214 and Bi‑214 to infer Ra‑226 activity, in accordance with ISO 18589-2, which governs environmental radioactivity measurements [8]. To ensure radon tightness during storage, samples were placed in hermetically sealed containers with screw-top lids and PTFE-lined seals. Each laboratory verified radon retention using standard reference materials, confirming that secular equilibrium was reliably achieved prior to analysis.

### Gamma spectrometry measurements

(a) GeLi HP detector—University of Cantabria (UC)

**Fig. 2:**
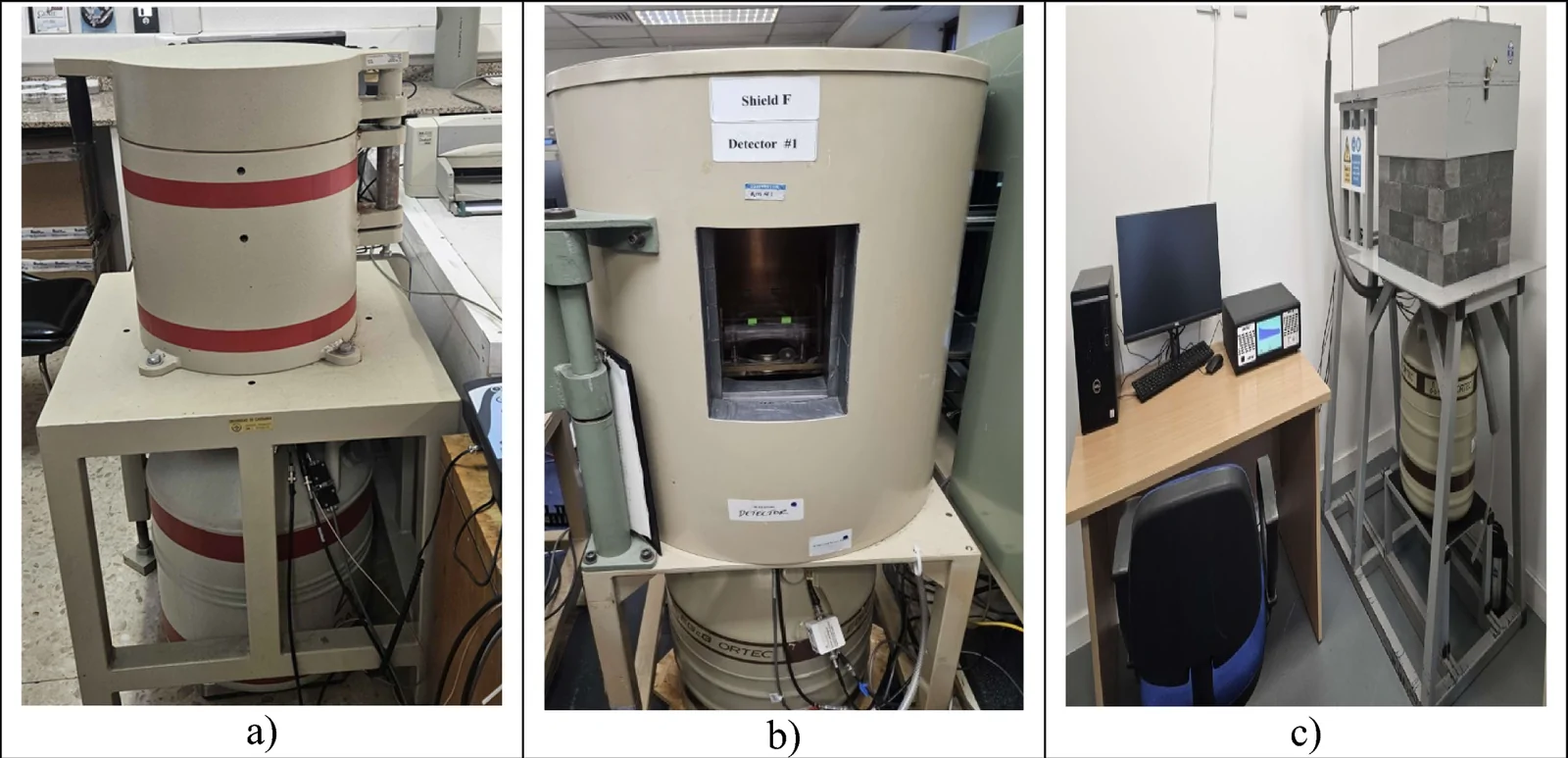
Gamma spectrometry systems used in this study: **a** University of Cantabria (GeLi HP), **b** EPA Laboratory (HPGe), **c** University College Dublin (HPGe)

The initial set of gamma spectrometry measurements was performed at the University of Cantabria, Spain, using a lithium-drifted high-purity germanium detector (GeLi HP) configured in a planar geometry (Fig. 2a). The detector was manufactured by Canberra (Model: 7229-3020), with a relative efficiency of 20% [9]. It was operated in a low-background environment and calibrated for absolute full-energy peak efficiency based on the specific sample geometry used in this study.

The detector’s ability to detect gamma rays change depending on the energy of the radiation and the sample geometry. For example, a typical efficiency was 5% for Am-241 (59.5 keV), 0.9% for Cs-137 (662 keV), and 0.4% for K-40 (1460 keV). This shows the detector could measure a wide range of gamma-ray energies, from low to high.

Furthermore, the detector possess excellent energy resolution, critical for distinguishing between different radionuclides in the sample. The resolution was less than 1% for both Cs-137 and Co-60, corresponding to an absolute energy resolution of 0.45 keV at 59.5 keV and 1.90 keV at 1332 keV, respectively.

Measurements covered gamma energies from 30 to 3000 keV. Samples were placed in 250 ml GEO geometry containers and measured for 2 h. Prior to measurement, samples were sealed and stored for 28 days to allow for secular equilibrium to be established.

The detector system was calibrated using standard sources prepared in the laboratory. Data acquisition and analysis were conducted using Genie 2000 software, which also applied essential corrections for effects such as true coincidence summing and self-attenuation to improve measurement accuracy. True coincidence summing (TCS) corrections were applied using Genie 2000 based on known nuclear decay schemes, detector geometry, and emission probabilities. Self-attenuation corrections were estimated from sample composition and density, with attenuation coefficients validated through transmission measurements using low-energy gamma sources. This combined approach improved accuracy, especially at low energies where attenuation effects are significant.

(b) HPGe detector—EPA Laboratory

Supplementary gamma spectrometry measurements were performed at the Environmental Protection Agency (EPA) Laboratory using a high-purity germanium (HPGe) detector configured in planar geometry with a relative efficiency of 70% (Fig. 2b). The detector was manufactured by ORTEC (Ametek Advanced Measurement Technology, USA), with model number GMX-7023-S and serial number 41-N31433A. It exhibited excellent energy resolution, with a full width at half maximum (FWHM) of 122 eV at 14.4 keV (Co-57) and 1.22 keV at 1332 keV (Co-60), enabling high-resolution detection across a broad gamma energy spectrum.

Sample aliquots were sealed in 250 ml cylindrical containers (250 GEO geometry) and measured over a live counting period of 24 h. Prior to measurement, samples were stored for a minimum of 30 days to allow for secular equilibrium.

Efficiency calibration was carried out using certified multi-gamma reference standards (NPL R08-02-2022060255-1) covering the energy range of 59–1836 keV. Correction factors for true coincidence summing and self-attenuation were determined using the Gespecor V4 software using generic compositions for the materials analyzed, and the efficiency curves were corrected for TCS effects prior to being approved for use. Spectrum acquisition and data processing were conducted using Genie 2000 (v3.4) with Apex-Gamma analysis modules.

(c) HPGe detector—University College Dublin (UCD)

The third set of gamma spectrometry measurements was conducted at University College Dublin (UCD) using a high-purity germanium (HPGe) detector (Ortec GMX-15190-SV, Serial No. 24-N-19NB) with a relative efficiency of 19% at 1.33 MeV (Co-60) (Fig. 2c). The detector demonstrated excellent energy resolution, with a full width at half maximum (FWHM) of 862 eV at 122 keV (Co-57) and 1.90 keV at 1332 keV (Co-60), enabling precise identification and quantification of gamma-emitting radionuclides across a wide energy range. The system was operated using an Ortec DSPEC 50 digital signal processing unit and spectra analyzed using Ortec’s Gamma Vision analysis software.

Samples were sealed in 250 ml cylindrical containers (250 GEO geometry) and stored for a minimum of 30 days to allow for secular equilibrium between Ra-226 and its progeny. Each sample was counted for 72 h to ensure adequate counting statistics and improved measurement precision.

Efficiency calibration was performed using certified multi-gamma reference standards (NPL R08-02-2022060255-1). Efficiency and true coincidence summing corrections were applied using the GESPECOR and EFFTRAN software tools. For these, generic elemental compositions were tuned to match the self-attenuation of each sample, as determined using transmission experiments over a range of energies. All corrections and analyses were carried out in accordance with established protocols.

### Activity determination of Ra-226, Th-232, and K-40

The activity concentration *A(E*) of radionuclides in the samples, expressed in becquerels per kilogram (Bq·kg⁻^1^), was determined using the following equation [9, 10]:

1 $${\boldsymbol{A}}\left( {\boldsymbol{E}} \right) = { }\frac{{C_{net} }}{{\varepsilon \left( E \right){ } \times {\text{ I }} \times { }t{ } \times { }m}}$$

where

- C_net_: Net counts for a specific gamma line corrected for background,
- ε(E): Full-energy peak efficiency at energy *E*,
- I: Gamma emission probability of the gamma-ray decay at energy *E*,
- t: Measurement time in second,
- m: Sample mass (kg).

The activities of Ra-226 and Th-232 were determined indirectly through the analysis of gamma emissions from their respective decay progeny, assuming secular equilibrium within their decay chains. For Ra-226, gamma lines at 295.1 keV (19.2%) and 351.9 keV (37.1%) from Pb-214, along with 1764.5 keV (15.4%) from Bi-214, were used to minimize the effects of true coincidence summing for UC. At UCD, the 609.3 keV gamma line of Bi-214 (45.5%) was also employed for quantification due to its strong emission intensity and consistent spectral visibility. It is noted that the EPA protocol additionally includes the 1120 keV and 1764 keV lines along with the 609 keV line for Bi-214 determination.

For Th-232, activity is generally assessed using the 338.4 keV (12%) gamma emission from Ac-228 and the 238.6 keV (44.6%) line from Pb-212. At UCD, quantification was based solely on the 338.4 keV line from Ac-228. Although the presence of Pb-212 in the spectra suggested equilibrium along the decay chain, this line was excluded from quantification in this dataset. The EPA similarly uses multiple gamma lines of Ac-228 (338.4, 911, and 968 keV) for Th-232 determination, and for UC samples, the method is consistent with UCD, relying solely on the 338.4 keV line with verification via the 911 keV line.

Reported uncertainties correspond to one standard deviation and include counting statistics only. Other sources of uncertainty (e.g. efficiency calibration and sample effects) are not included.

The activity of K-40 was directly determined from its 1460.8 keV gamma emission, which has an emission probability of 10.7%. All full-energy peaks (FEP) used in this analysis were corrected for possible spectral interferences from overlapping peaks of other nuclides where necessary to ensure accurate quantification [11, 12].

### Gamma index (Iγ)

The gamma activity concentration index (Iγ) is used as a screening tool to assess the suitability of building materials with regard to radiological protection, particularly for gamma radiation exposure to occupants. It is recommended by the European Commission [2] and described in Radiation Protection Report No. 112 (RP 112) [2, 13]. The index is calculated as:

2 $${\mathbf{I\gamma }} \, = {\mathrm{A}}_{{{\mathrm{Ra}}}} /{3}00 + {\mathrm{A}}_{{{\mathrm{Th}}}} /{2}00 + {\mathrm{A}}_{{\mathrm{K}}} /{3}000$$

where: A_Ra_, A_Th_, and A_K_ are the specific activities of Ra-226, Th-232, and K-40, respectively, expressed in Bq·kg⁻^1^.

According to RP 112 and EC guidance, the index is interpreted as follows:

- Iγ ≤ 1: Suitable for use in bulk materials (e.g., concrete).
- Iγ > 1: Further assessment is required to ensure annual effective dose does not exceed 1 mSv. For superficially applied or limited-use materials (e.g., tiles or mortar), Iγ up to 6 may be acceptable.

The index provides a conservative estimate of gamma dose and does not account for all exposure pathways, such as radon inhalation. It is intended as a screening tool, whereas full compliance with radiation protection requirements is determined according to the EURATOM Basic Safety Standards Directive (2013/59/Euratom) [3], which is legally binding for EU member states.

## Results and discussion

High-resolution gamma spectrometry using the three detectors described earlier were used to determine the activity concentrations of Ra-226, Th-232, and K-40 in all samples. The results, summarized in Table 1, showed only minor variations between laboratories. To enhance reliability and capture potential inter-laboratory differences, gamma activity index calculations were performed separately for each dataset, as presented in Table 2.

**Table 1:** Activity concentrations of Ra-226, Th-232, and K-40 (Bq kg⁻^1^) in various building materials. Uncertainties correspond to one standard deviation and include counting statistics only

Sample code	Activity concentration (Bq kg^−1^)		
	A (^226^Ra)	A (^232^Th)	A (^40^ K)
New tilesᵃ	102 ± 1.80	71.0 ± 2.40	894 ± 31.0
New tilesᵇ	99.3 ± 1.50	69.0 ± 1.90	905 ± 29.0
New tilesᶜ	68.1 ± 6.20	76.0 ± 14.0	895 ± 84.0
New cementᵃ	33.0 ± 0.60	9.00 ± 0.40	205 ± 7.30
New cementᵇ	32.8 ± 0.50	10.1 ± 0.40	203 ± 7.00
New cementᶜ	30.8 ± 3.10	11.4 ± 3.10	179 ± 21.0
1964 concreteᵃ	36.0 ± 0.60	3.00 ± 0.30	50.0 ± 2.50
1964 concreteᵇ	35.6 ± 2.40	2.74 ± 0.20	52.0 ± 2.40
1964 concreteᶜ	28.8 ± 2.70	N.D	113 ± 14.0
World averageᵈ	50.0	50.0	500

^a^Measurement by EPA Laboratory (HPGe detector)

^b^Measurement by University College Dublin (HPGe detector)

^c^Measurement by University of Cantabria (GeLi HP detector)

^d^World-Average Activity Concentrations in Building Materials (UNSCEAR) [1]

**Table 2:** Gamma Index (Iγ) values for selected building materials

Sample code	Gamma index (Iγ)
New tilesᵃ	0.99
New tilesᵇ	0.98
New tilesᶜ	0.91
New cementᵃ	0.22
New cementᵇ	0.23
New cementᶜ	0.22
1964 concreteᵃ	0.15
1964 concreteᵇ	0.15
1964 concreteᶜ	0.13

^a^Measurement by EPA Laboratory (HPGe detector)

^b^Measurement by University College Dublin (HPGe detector)

^c^Measurement by University of Cantabria (GeLi HP detector)

### Radionuclide activities in building materials

Table 1 presents the activity concentrations of Ra-226, Th-232, and K-40 for each material and laboratory. New tile samples consistently exhibited higher concentrations of all three radionuclides compared to cement and demolition of concrete sourced from a 1964 residential building. Th-232 activity in both the cement and the 1964 concrete was often negligible or below detection limits.

The Ra-226 concentrations in all tile samples exceeded the world average [1], ranging from 68.1 ± 6.20 Bq kg⁻^1^ to 102 ± 1.80 Bq kg⁻^1^, indicating a significant presence of uranium series radionuclides. In contrast, cement samples showed lower Ra-226 levels, between 30.8 ± 3.10 and 33.0 ± 0.60 Bq kg⁻^1^, with demolition concrete displaying comparable concentrations ranging from 28.8 ± 2.70 to 36.0 ± 0.60 Bq kg⁻^1^. These findings were further confirmed by U-238 concentrations measured via Th-234, suggesting a relatively good equilibrium between U-238 and Ra-226 in the samples.

Th-232 activity was considerably lower in all cement and concrete samples, often below detection limits. Only the tile samples, particularly those analyzed by the University of Cantabria, showed elevated Th-232 levels (up to 76.0 ± 14.0 Bq kg⁻^1^), exceeding the global average. However, it should be noted that, within the uncertainties, the Th-232 concentrations in tile samples from the University of Cantabria, EPA, and UCD are consistent and not significantly different. This suggests that ceramic materials may incorporate more thorium than cement or older concrete. The elevated Th-232 concentrations in tiles may be attributed in part to the glazing layer, which is known to sometimes contain naturally occurring radionuclides such as Th-232 and K-40, depending on the raw materials used in manufacture [14, 15].

K-40 activity concentrations varied significantly across sample types. The highest values were observed in new tile samples, around 900 Bq kg⁻^1^, well above the world average of 500 Bq kg⁻^1^. In contrast, new cement and demolition concrete samples had much lower K-40 activity, ranging from 50.0 ± 2.50 to 205 ± 7.30 Bq kg⁻^1^. One notable exception was the concrete sample measured by the Cantabria laboratory, which exhibited a K-40 level of 113 ± 14.0 Bq kg⁻^1^.

**Fig. 3:**
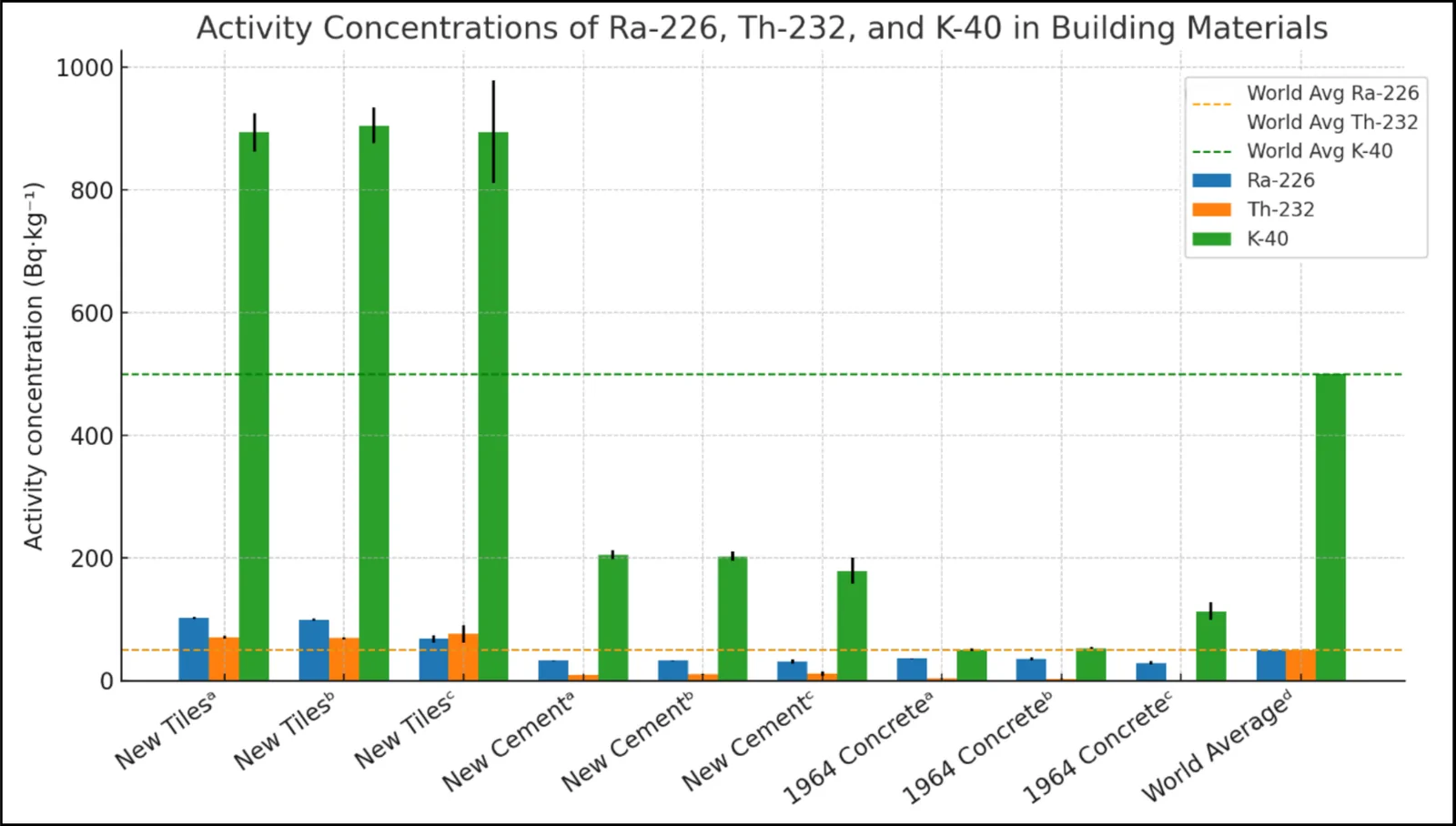
Comparative activity concentrations of Ra-226, Th-232, and K-40 in tiles, cement, and demolition of concrete, as measured by three independent laboratories

As shown in Fig. 3, radionuclide concentrations vary markedly between tiles and cementitious materials. This difference is primarily due to the distinct mineralogical origins of their raw materials. Tiles, derived from clay-rich sediments, naturally contain higher levels of uranium- and potassium-bearing minerals. Conversely, cement and concrete are mainly composed of limestone and silicate materials, which typically exhibit lower natural radioactivity [16, 17].

The inter-laboratory variations observed remained within acceptable experimental uncertainties, defined here as relative differences of less than 10% for Ra-226 and K-40, and up to 20% for Th-232 due to its lower activity levels in some materials. These variations fall within the expected range for gamma spectrometry of solid building materials, confirming the robustness and consistency of the measurement protocol across different laboratories and detector types. These findings emphasize the importance of conducting systematic radiological assessments of building materials, especially in contexts involving prolonged indoor exposure, to ensure compliance with safety regulations.

### Assessment of gamma index (Iγ)

**Fig. 4:**
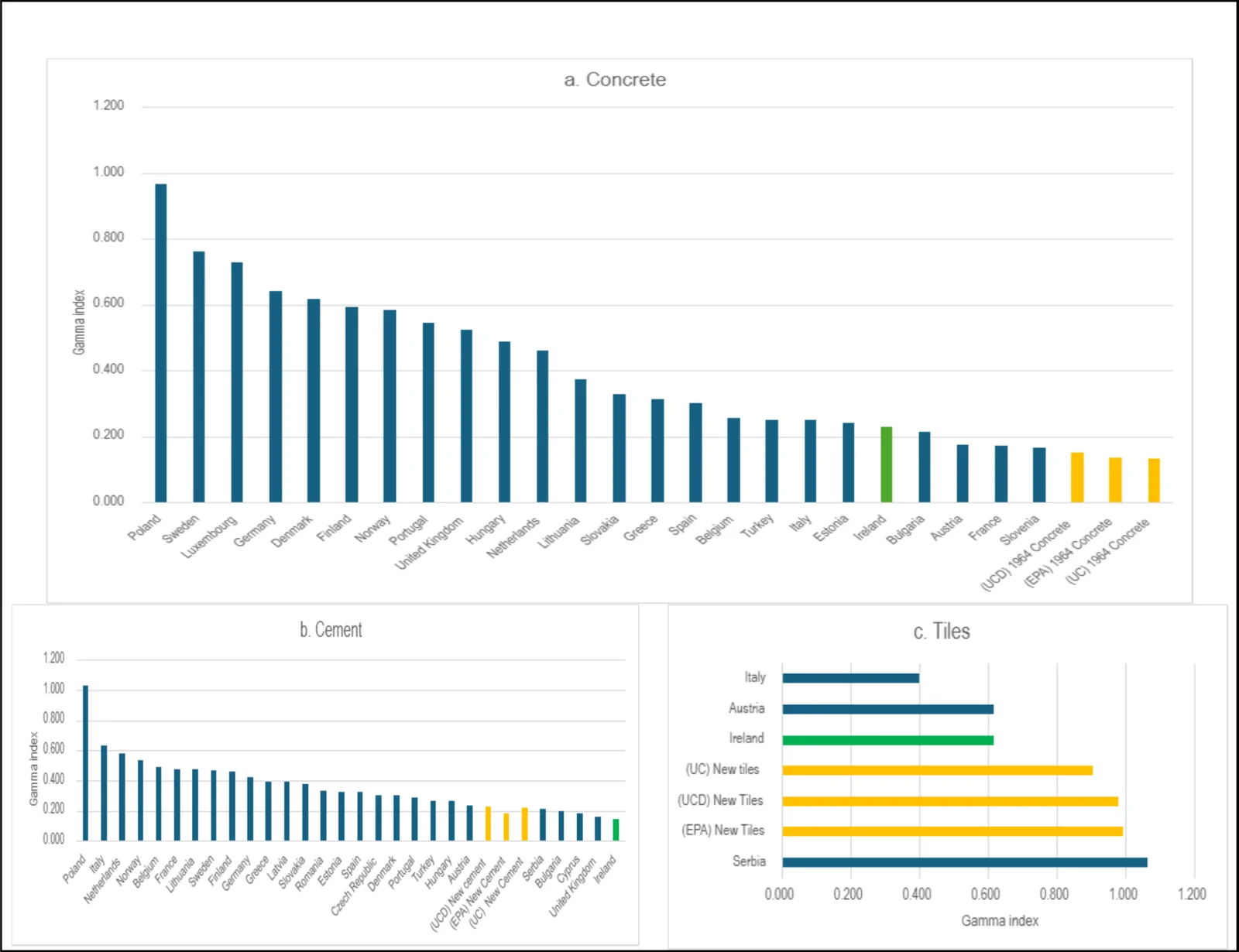
Comparative Gamma Activity Index (Iγ) values for concrete (**a**), cement (**b**), and tile (**c**) samples in this study versus published data from European countries

The Gamma Activity Index (Iγ) for each building material was calculated based on the activity concentrations of Ra-226, Th-232, and K-40, using Eq. (2). The results are presented in Table 2 and visualized in Fig. 4. Measurements were independently conducted by three laboratories using high-resolution gamma spectrometry, ensuring methodological consistency and data reliability.

New tile samples exhibited the highest Iγ values, ranging from 0.91 to 0.99, approaching the recommended screening threshold of 1. These elevated values reflect the higher concentrations of Ra-226, Th-232, and K-40 detected in tile samples and are attributed to the clay-rich raw materials used in their production, which naturally contain uranium- and potassium-bearing minerals. In contrast, new cement samples showed significantly lower gamma indices (0.22–0.23), well below the safety threshold. This reflects their lower radionuclide content, attributed to the predominance of limestone and silicate minerals in their composition. As a result, cement poses minimal gamma radiation risk under normal usage conditions.

Demolition concrete samples recorded the lowest gamma index values, between 0.13 and 0.15, reaffirming the low radiological hazard associated with older concrete. These results also support the potential reuse of such materials, aligning with sustainability goals and circular economy principles in the construction sector.

Uncertainties in Iγ, derived from counting statistics, are small and do not affect regulatory interpretation. Tile samples approach the threshold (Iγ ≈ 1) but remain within acceptable limits, although some caution may be warranted for extensive indoor use. Under the EURATOM Basic Safety Standards Directive (2013/59/Euratom) [3], these values do not require further dose assessment for bulk materials under normal conditions.

## Comparison with published data from European studies

To assess the regional relevance of the results, the Gamma Activity Index (Iγ) values measured for Irish building materials in this study were compared with published data from various European countries. The comparative analysis includes concrete, cement, and tile samples, as illustrated in Fig. 4a–c.

For Irish demolition concrete materials dating from 1964, the Iγ values ranged from 0.13 to 0.15, among the lowest reported across Europe. In contrast, significantly higher values have been recorded in countries such as Poland, Romania, and the Czech Republic, where gamma indices exceed 1.0 [5, 18]. These differences likely reflect geological and mineralogical variations, particularly the use of aggregates enriched in naturally occurring radionuclides. The low indices observed in our samples underscore their radiological safety and support their potential for reuse in sustainable construction aligned with circular economy principles.

Regarding cement, the newly produced samples exhibited gamma index values between 0.18 and 0.23, well below the recommended threshold. These results are consistent with data reported from Ireland, the United Kingdom, and Cyprus [19]. In contrast, higher values sometimes approaching or exceeding 0.8 have been observed in France, Italy, and Poland [20, 21].

For tiles, gamma index values ranged from 0.91 to 0.99, approaching the European Commission’s recommended upper limit for building materials. These values are comparable to those reported in Serbia, Austria, and Ireland [22, 23]. Although still within acceptable safety limits, these findings highlight the necessity for material-specific radiological control, particularly for tiles intended for large-scale indoor applications. Overall, this cross-national comparison emphasizes the importance of regional screening and thorough material characterization to ensure radiological safety in the built environment.

## Conclusion

This study presents a radiological assessment of commonly used building materials in Ireland, focusing on the activity concentrations of naturally occurring radionuclides (Ra-226, Th-232, and K-40) and their contribution to indoor gamma radiation, as evaluated through the Gamma Index (Iγ). High-resolution gamma spectrometry was independently performed in three laboratories, each utilizing distinct detector systems and calibration procedures. The strong inter-laboratory agreement confirms the robustness, accuracy, and reproducibility of the activity concentration measurements across all material types, including tiles, cement, and demolition concrete.

The results indicate that tile samples consistently exhibited the highest concentrations of all three radionuclides, yielding Iγ values close to the European Commission’s recommended screening threshold of 1.0. In contrast, newly produced cement and 1964 concrete samples showed substantially lower radionuclide activities, with corresponding Iγ values well below the regulatory threshold. These materials can therefore be considered radiologically safe for conventional construction applications. However, the elevated radioactivity levels in certain tile products highlight the need for targeted radiological oversight, particularly for materials intended for extensive indoor use.

The study underscores the critical role of raw material origin and mineralogical composition in influencing natural radioactivity levels in building products. Additionally, the demonstrated inter-laboratory consistency reinforces gamma spectrometry as a reliable and standardized method for radiological monitoring and compliance.

By establishing baseline data and validating methodological reliability, this work contributes to the development of a national framework for the radiological screening of building materials in Ireland. The findings also support broader European efforts related to radiological protection, sustainable construction practices, and the advancement of circular economy principles in the built environment.

## Data Availability

Manuscript data is available on request.
